# Multiparametric Classification of Skin from Osteogenesis Imperfecta Patients and Controls by Quantitative Magnetic Resonance Microimaging

**DOI:** 10.1371/journal.pone.0157891

**Published:** 2016-07-14

**Authors:** Beth G. Ashinsky, Kenneth W. Fishbein, Erin M. Carter, Ping-Chang Lin, Nancy Pleshko, Cathleen L. Raggio, Richard G. Spencer

**Affiliations:** 1 Laboratory of Clinical Investigation, Magnetic Resonance Imaging and Spectroscopy Section, National Institute on Aging, National Institutes of Health, Baltimore, Maryland, United States of America; 2 Kathryn O. and Alan C. Greenberg Center for Skeletal Dysplasias, Hospital for Special Surgery, New York, New York, United States of America; 3 Core Imaging Facility for Small Animals, GRU Cancer Center, Augusta University Augusta, Georiga, United States of America; 4 Department of Bioengineering, College of Engineering, Temple University, Philadelphia, United States of America; 5 Department of Orthopaedics, Hospital for Special Surgery, New York, New York, United States of America; Banner Alzheimer's Institute, UNITED STATES

## Abstract

The purpose of this study is to evaluate the ability of quantitative magnetic resonance imaging (MRI) to discriminate between skin biopsies from individuals with osteogenesis imperfecta (OI) and skin biopsies from individuals without OI. Skin biopsies from nine controls (unaffected) and nine OI patients were imaged to generate maps of five separate MR parameters, T_1_, T_2_, k_m_, MTR and ADC. Parameter values were calculated over the dermal region and used for univariate and multiparametric classification analysis. A substantial degree of overlap of individual MR parameters was observed between control and OI groups, which limited the sensitivity and specificity of univariate classification. Classification accuracies ranging between 39% and 67% were found depending on the variable of investigation, with T_2_ yielding the best accuracy of 67%. When several MR parameters were considered simultaneously in a multivariate analysis, the classification accuracies improved up to 89% for specific combinations, including the combination of T_2_ and k_m_. These results indicate that multiparametric classification by quantitative MRI is able to detect differences between the skin of OI patients and of unaffected individuals, which motivates further study of quantitative MRI for the clinical diagnosis of OI.

## Introduction

Osteogenesis imperfecta (OI) is a heritable connective tissue disorder characterized by increased bone fragility and predisposition to fractures [1]. Extra-skeletal involvement may include the integumentary, cardiovascular, pulmonary, and/or ocular systems [2]. Although genetically heterogeneous, OI is caused primarily by dominant mutations in the COL1A1 or COL1A2 genes, which encode the pro-α1 and pro-α2 chains of type I collagen, respectively [1–4]. Originally, OI was classified by Sillence *et al*. [5] into four types (I-IV) based on phenotype; the mildest form, type I, results in few fractures, blue sclerae and normal life expectancy [6]. Type II is the most severe form of OI and results in perinatal death, while types III and IV are characterized by progressive and moderate deformity. More recently, novel types of OI (V-X) due to mutations of non-collagen genes have been described based on clinical and histological features [2]. Because the current study includes two subjects with type IX, we specifically note that this phenotype results from a defect in the cyclophilin B protein of the prolyl 3-hydroxylase complex, which alters the post-translational modification and folding of type I collagen [7]. Individuals with type IX OI present with moderate to severe phenotypes, similar to types III and IV.

Diagnosis of OI is based on clinical and radiological criteria, and increasingly through molecular genetic testing. While severe OI phenotypes can be straightforward to identify, the diagnosis of milder phenotypes is more difficult, relying upon confirmatory mutation analysis. However, timely diagnosis of milder phenotypes may be of particular clinical importance since unexplained fractures in infants can be characteristic of either OI or non-accidental injury [8].

Skin analysis has been of particular interest in animal models and patients with connective tissue disorders [9]. Skin from transgenic mouse models of types I and II OI [6, 10] and from human patients with mild and severe OI [1] has been examined to identify characteristic chemical and structural features present in the dermal collagen. Histochemical collagen secretion studies of the skin have indicated a decrease in overall collagen content, while electron and non-linear microscopic studies have shown a thinning of the dermal layer and compromised collagen packing and density [1, 11]. Another study used a suction cup technique to evaluate the mechanical properties of skin in patients with mild and moderate-to-severe OI (types I and III), and found significant abnormalities in skin elasticity, distensibility and hysteresis compared to controls [12]. Most recently, Balasubramanian et al. demonstrated that histological and electron microscopic findings in skin biopsies from OI patients, such as increased collagen fibril diameter variability, may indicate an increased likelihood of finding a mutation in type I collagen genes [13]. Thus, the possibility of investigating skin manifestations of the molecular changes associated with OI is of particular interest.

Magnetic resonance imaging (MRI) is a noninvasive modality that can assess a wide range of musculoskeletal anatomy at the tissue level [14, 15]. Our previous work on OI manifestations in skin investigated the biophysical effects of OI using MRI of skin from a murine model of moderate-to-severe OI, the *oim/oim* mouse [16], phenotypically similar with type III OI in humans. We found that MR parameters, including transverse relaxation time (T_2_) and magnetization transfer rate (k_m_), reflected variations in collagen content and packing in the skin of the *oim/oim* and wild type mice [16]. In the *oim/oim* model, a collagen-depleted lower dermal layer was observed with k_m_ values 50% lower and T_2_ values 30% greater than controls. In addition to T_2_ and k_m_, other MR parameters, including MTR (magnetization transfer ratio), T_1_ (longitudinal relaxation), and ADC (apparent diffusion coefficient), have been explored in other skin studies [17].

Previously, we reported several studies using similar MR modalities with univariate and multivariate classification to distinguish normal from pathomimetically-digested cartilage, another collagen-rich tissue [18–20]. We observed improved classification results through multiparametric techniques using Gaussian clustering and support vector machine (SVM) models. In the present study, we likewise apply univariate and multivariate SVM classification, specifically tuned for this dataset, to investigate the use of MR parameters to distinguish skin from OI patients from that of control subjects.

## Methods

### Human skin sample acquisition

This study was performed under a protocol approved by the IRB of the Hospital for Special Surgery, New York, NY. All subjects completed a written informed consent form or assent form when applicable. Parents or legal guardians completed a written informed consent form for all minors enrolled in the study, and minors seven years or older completed a written assent form. All consent and assent forms were IRB approved. Documents were stored in the regulatory binder for the IRB study protocol and participants received copies of the forms for personal record.

Skin samples were taken from nine subjects (age range 3–45 years; three female, six male) with OI based on clinical and radiographic criteria with confirmation by molecular genetic testing (Table 1). Samples were likewise taken from nine control subjects (age range 3–55 years; five female, four male).

**Table 1:** OI and Control patient characteristics.

OI type	Disease Severity	OI Age (years)	OI Gender	Control Age (years)	Control Gender
Type I	Mild	37	Male	3	Male
Type I	Mild	40	Male	5	Male
Type III	Severe	21	Male	8	Male
Type III	Severe	23	Female	30	Female
Type IV	Moderate	25	Male	33	Male
Type IV	Moderate	45	Male	33	Male
Type IV	Moderate	17	Female	46	Female
Type IX	Moderate	9	Male	53	Male
Type IX	Moderate	3	Female	55	Female

Full-thickness skin biopsies were collected from the volar aspect of each subject's forearm using a 3 mm dermal punch (Acu-Punch kit, Acuderm, Inc. Ft. Lauderdale, FL, USA) under local anesthetic. After collection, specimens were immediately placed into a mesh CellSafe biopsy insert (Electron Microscopy Sciences, Hatfield, PA, USA), housed inside a biopsy cassette, and submerged in DPBS 1X buffer containing Sigma P-2714 protease inhibitor (PI) and 12.5 mM GM6001 MMP inhibitor (EMD Millipore, Darmstadt, Germany), adjusted to pH 7.5, where they remained during MRI data acquisition.

### Histology

Biopsies were processed for histology after MRI data collection. Tissues were fixed in 70% ethanol, paraffin embedded, sectioned at 5 microns thickness, and stained with picrosirius red to visualize collagen and overall tissue morphology.

### MRI protocol

Samples were scanned using a Bruker 9.4 T DMX microimaging spectrometer equipped with a 10 mm birdcage RF coil and 1000 mT/m shielded gradients. Samples were loaded so that the B_0_ field was approximately perpendicular to the epidermal surface. Temperature was maintained at 4.0 ± 0.1°C during MRI experiments using chilled air from a vortex tube (Exair, Inc., Cincinnati, OH, USA) with modulated reheating by the spectrometer temperature control system. All images were acquired using single-slice spin echo sequences with field of view (FOV) = 0.25 × 1.2 cm (perpendicular × parallel to epidermis), matrix size (MTX) = 128 × 256, in-plane resolution = 19.5 microns × 46.9 microns and slice thickness = 400 microns. We selected 5 of the most commonly employed MR outcome measures for tissue characterization.

#### T_2_ measurements

T_2_-weighted images were acquired using a multiecho CPMG sequence with echo time (TE) = 5.3 ms, 64 echoes, number of excitations (NEX) = 8, repetition time (TR) = 1.3 s and total scan time = 44 minutes.

#### T_1_ measurements

T_1_-weighted images were acquired using a single echo sequence with TR ranging from 0.1 s to 15 s in 12 increments, NEX = 4, TE = 7.2 ms and total scan time = 5 hours and 10 minutes.

#### k_m_ and MTR measurements

Magnetization transfer-weighted images were acquired using a single-echo sequence with variable-duration off-resonance presaturation at an offset of +6000 Hz from H_2_O, B_1_ 12 mT, presaturation pulse length = 100 ms to 4.6 s in 8 increments, NEX = 4, TR 5 s, TE = 7.2 ms and total scan time = 5 hours and 42 minutes.

#### ADC measurements

Diffusion-weighted images were acquired using a spin echo sequence incorporating standard Stejskal-Tanner diffusion-sensitizing gradients with d = 5 ms; D = 10.25 ms, TE = 19.4 ms, diffusion gradient strength = 0 to 900 mT/m in 9 increments, NEX = 8, TR = 1 s and total scan time = 5 hours and 7 minutes. This scan was performed with the diffusion-sensitizing gradient oriented parallel to B_0_ resulting in b = 473 to 16880 s/mm^2^.

### Parameter maps and region of interest selection

**Fig 1:**
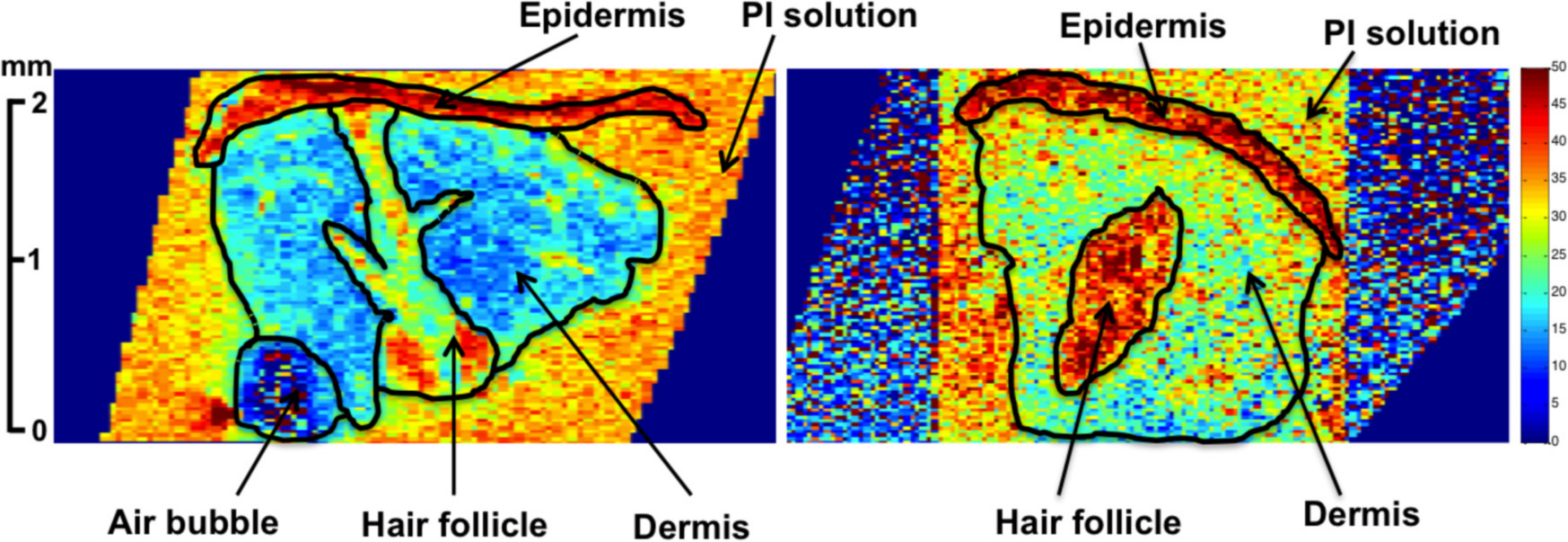
T_2_ parameter maps of control (a) and OI (b) skin samples. Regions of interest are outlined and labeled. The static magnetic field, B_0_, is oriented vertically. Parameters for analysis were obtained by averaging over the entire dermal region.

For all parameters, maps were generated from magnitude MR images using a pixel-wise nonlinear least squares fit to the appropriate monoexponential function including a constant offset term. Apparent magnetization transfer rate, k_m_, was derived by measuring signal intensity as a function of off-resonance saturation time and MTR was defined as 1 –(M_ss_/M_0_), where M_ss_ is the magnetization measured in the steady state and M_0_ is the equilibrium magnetization [21]. For each sample, the dermal ROI was manually drawn (Fig 1). The epidermis was distinguished from the dermis by the much shorter T_2_ and smaller ADC of the latter. Similarly, the interface between the dermis and the surrounding PBS/PI solution, as well as the margins of the hair follicle (if present) could be visualized on T_2_ and diffusion maps. The dermis ROI drawn on the T_2_ map of each sample was copied to all other parameter maps for that sample to verify its alignment with the anatomical structures visualized on each map.

### Classification Methods and Statistical Analysis

For each univariate or multivariate method, classification sensitivity and specificity were calculated. Sensitivity is the proportion of correctly assigned OI samples, specificity is the proportion of the correctly assigned control samples, and accuracy is the proportion of correctly assigned samples overall; for a balanced data set, this is the average of the sensitivity and specificity. All uni- and multivariate analyses were performed using in-house designed scripts based on the LIBSVM library [22] with the e1071 package [23], written in the R language. A two-tailed unpaired Student’s t-test was performed to test for statistically significant mean parameter differences between OI and control samples, and for gender differences in MR parameters, with p ≤ 0.05 considered significant. A Pearson correlation was performed to assess correlations between age and MR parameters, and the R values for strength of correlation and p values for significance evaluated. Analysis of variance (ANOVA) was performed to assess differences among MR parameters for the three OI patient groups, mild, moderate and severe.

#### Univariate classification

Univariate classification according to the Mahalanobis distance metric [24] was performed using the average values of the MR parameters for the dermis of each sample. A standard leave-one-out analysis was conducted in which each sample in the dataset was in turn designated as a validation sample, with the classification rule developed for the remaining samples, designated as the training set. The validation sample was then classified as “control” or “OI”. The Mahalanobis distance classification rule is based on the difference in arithmetic means of a specified MRI parameter normalized by group standard deviations (SD); this accounts for the fact that a larger standard within a group renders more likely a given deviation from the mean value [24]. Note that this approach to classification is independent of the underlying data distribution, but permits incorporation of unequal group standard deviations. This procedure was repeated for each of the 18 samples. Sensitivity, specificity and accuracy of classification using the specified MRI parameter were then calculated based on the results for the 18 samples.

#### Multivariate classification

Multivariate SVM classification analysis [20, 25] was performed on the full dataset (S1 Table). Briefly, the SVM algorithm solves a convex optimization problem by performing a nonlinear transformation of data points, in this case MRI parameter values, into a higher dimensional feature space in which a hyperplane is defined that provides the maximum separation between the “control” and “OI” classes with misclassifications permitted according to a pre-defined penalty. Further details of the SVM, including the use of the Gaussian radial basis kernel function and the Lagrangian dual function formalism, may be found in the standard literature [26] and in our recent work in classification of degraded cartilage [20]. Individual MR parameter values were normalized by their group SDs prior to calculating the SVM models. Optimal parameter values for the breadth of the radial basis function and for the misclassification penalty were defined by a search over an exponentially-spaced grid over the ranges [22^−6^, 23] and [2^−1.5^, 2^4^], respectively. A standard leave-one-out analysis, as described previously, was performed for each parameter combination. Sensitivity, specificity and accuracy were again calculated after classifying individual samples according to the associated SVM model for each multiparametric combination. We note that the SVM approach, which is minimally dependent on data structure, was chosen for this work since the limited number of data points precluded any realistic attempt to determine the statistical distribution of the data set.

## Results

### Histology

**Fig 2:**
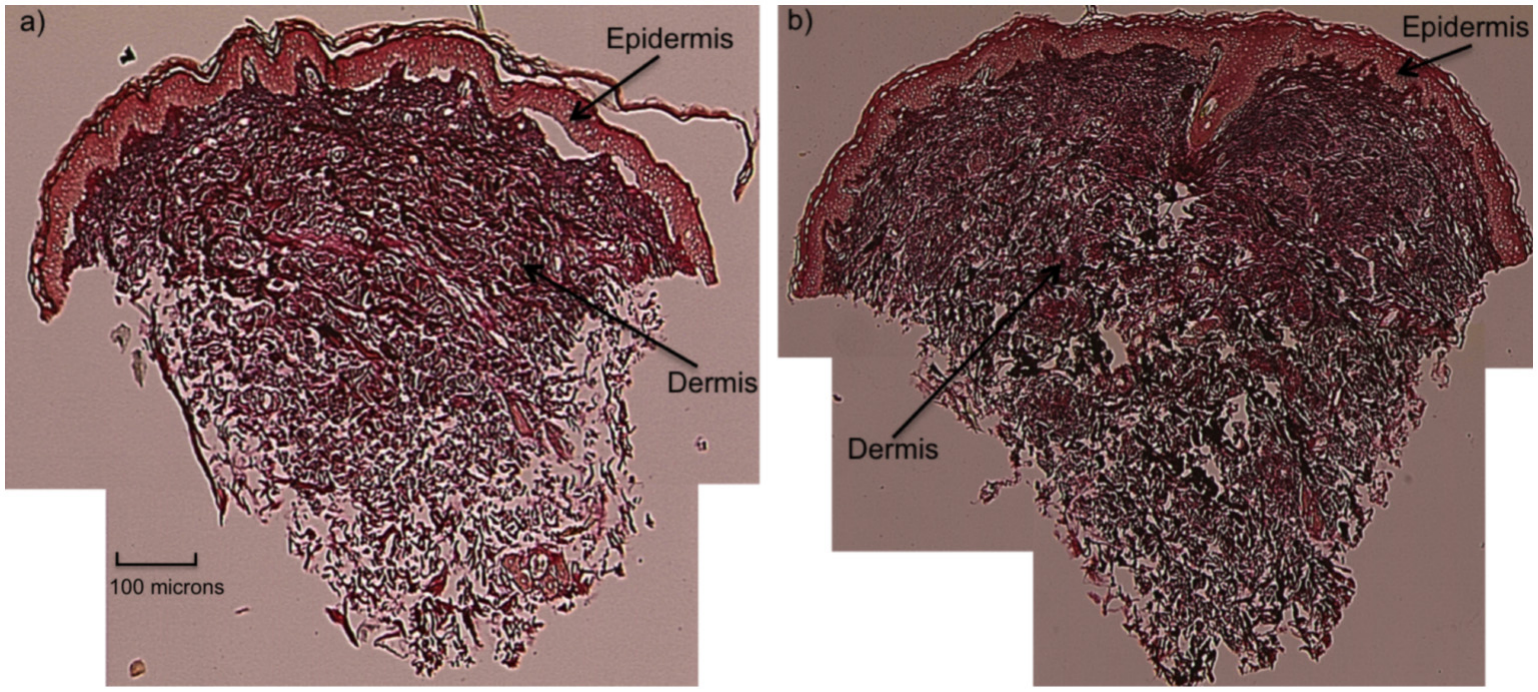
Representative histology samples from a control (a) and OI (Type I) (b) biopsies. Arrows indicate distinct epidermal and dermal layers in both tissues.

Fig 2 shows representative histology from control and OI (Type I) biopsies. Both tissues exhibit distinct epidermal and dermal layers. Qualitative differences in structural features between OI and control tissues were not obvious.

### Univariate analysis

**Fig 3:**
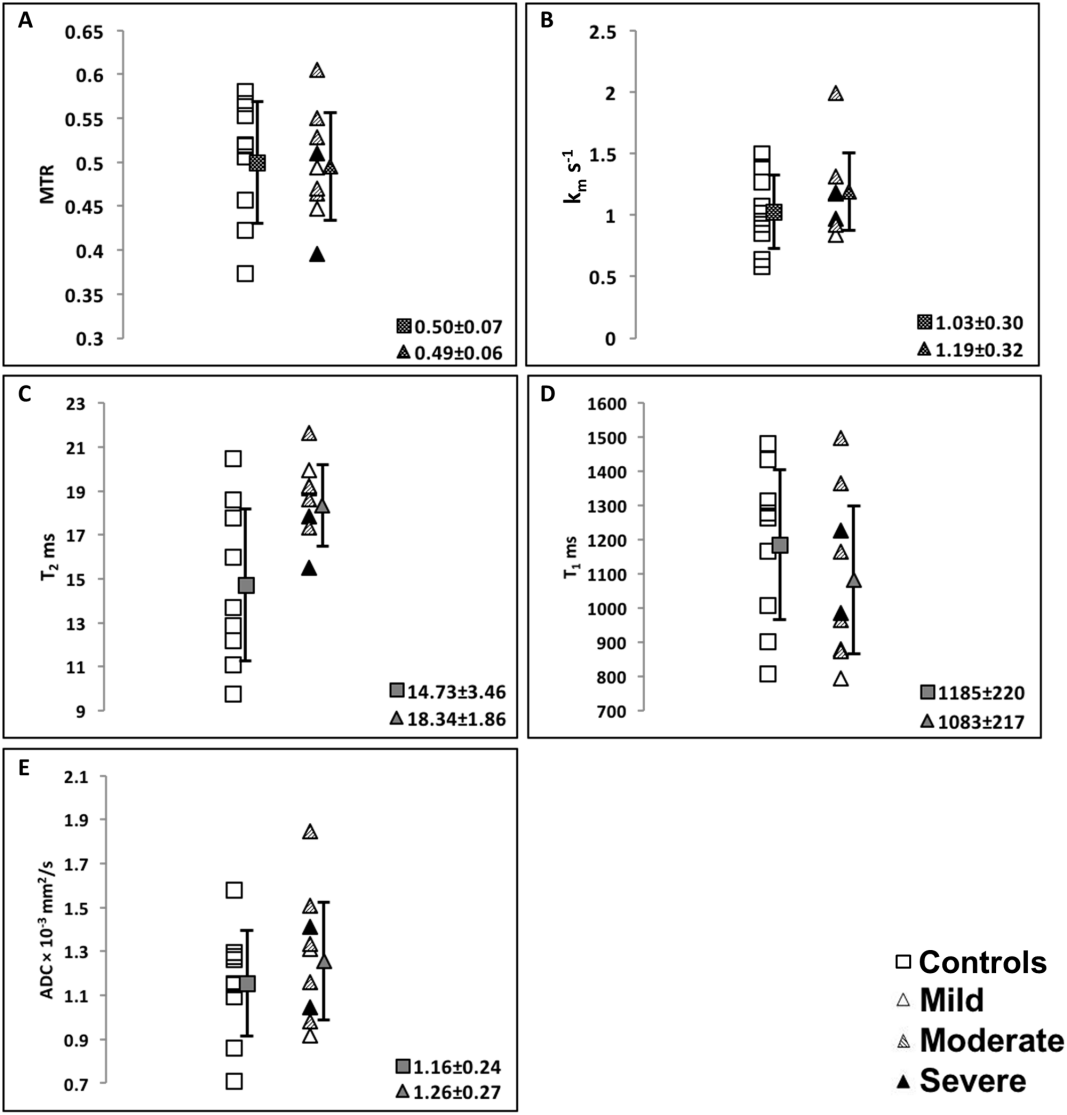
Average MR parameter values. Control subjects are represented as open squares and OI subjects are represented as shaded triangles with their corresponding group mean values. Error bars represented as solid squares and triangles, respectively, for A) MTR (control vs OI: p = 0.88), B) k_m_ (control vs OI: p = 0.32), C) T_2_ (control vs OI: p = 0.01), D) T_1_ (control vs OI: p = 0.38), E) ADC (control vs OI: p = 0.37). Note the high degree of overlap between groups for each parameter. Further, there is no trend for the OI values based on degree of severity for any MR parameter.

**Fig 4:**
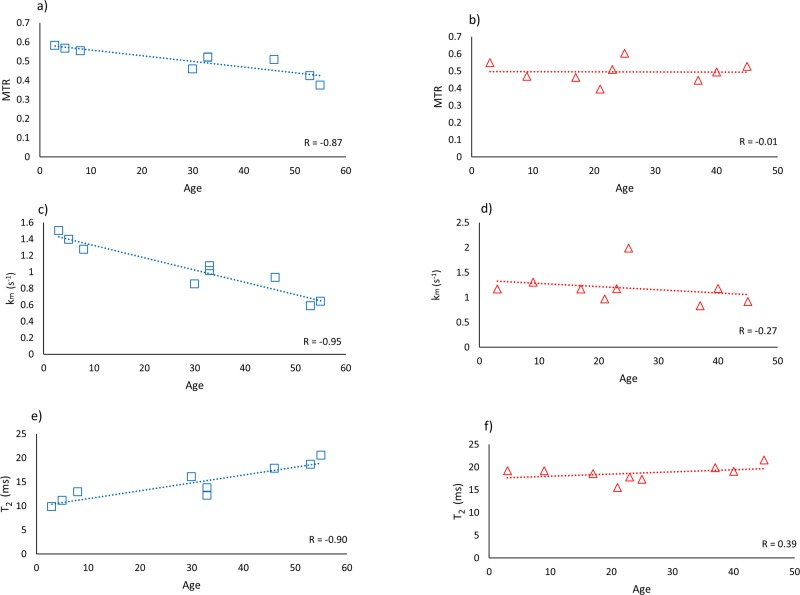
MTR, k_m_, and T_2_ plotted as a function of age for control (left column; blue symbols) and OI (right column; red symbols) subjects. Note that the pattern of correlations between MR parameters and age seen in control subjects is not present in the OI subjects.

Fig 3 shows the arithmetic means of the MR parameter values over all pixels in the dermal ROI for each subject, as well as overall group means and SDs. There were no statistically significant differences in MR parameters between genders for all subjects combined (MTR: p = 0.82, k_m_: p = 0.96, T_2_: p = 0.35, T_1_: p = 0.57, ADC: p = 0.10), with similarly nonsignificant differences between genders within control and OI groups. Likewise, there were no significant differences among the three groups of OI severity (for ANOVA between groups, MTR: p = 0.35, k_m_: p = 0.54, T_2_: p = 0.16, T_1_: p = 0.28, ADC: p = 0.63). In addition, no statistically significant correlations were found in the OI group between age or OI severity and any MR parameter. However, in the control subjects, a significant correlation was found between age and MTR (R = -0.87), k_m_ (R = -0.95), and T_2_ (R = -0.90) (p<0.01 in all cases; Fig 4).

T_2_ was the only parameter with a statistically significant difference (p = 0.0098) in the arithmetic means between the control and OI groups. For the other parameter comparisons between the Control and OI groups, the p values were: MTR, p = 0.88; k_m_, p = 0.32; T_1_, p = 0.38; ADC, p = 0.37. A substantial degree of overlap between control and OI groups is observed for each MR parameter, limiting the sensitivity and specificity of univariate classification (Table 2). MTR and ADC were the weakest performing parameters for univariate discrimination, yielding overall accuracies of 39% and 44%, respectively (Table 2). Use of T_2_ resulted in appreciably higher classification accuracy with a sensitivity, specificity and overall accuracy of 67%. The importance of the fact that the Mahalanobis distance accounts for differences in group SDs is clearly seen for T_2_, for which the SD of the mean of the control group was 3.46, while the SD of the mean of the OI group was 1.86 (Fig 2).

**Table 2:** Univariate classification results for the training and test sets using the Mahalanobis distance metric for each MR parameter.

MR		Training set			Test Set	
parameter	Sensitivity	Specificity	Accuracy	Sensitivity	Specificity	Accuracy
MTR	0.32	0.84	0.58	0.11	0.67	0.39
K_m_	0.63	0.67	0.65	0.56	0.67	0.61
T_2_	0.78	0.67	0.73	0.67	0.67	0.67
T_1_	0.56	0.67	0.61	0.56	0.67	0.61
ADC	0.57	0.55	0.56	0.44	0.44	0.44

### Multivariate analysis

**Fig 5:**
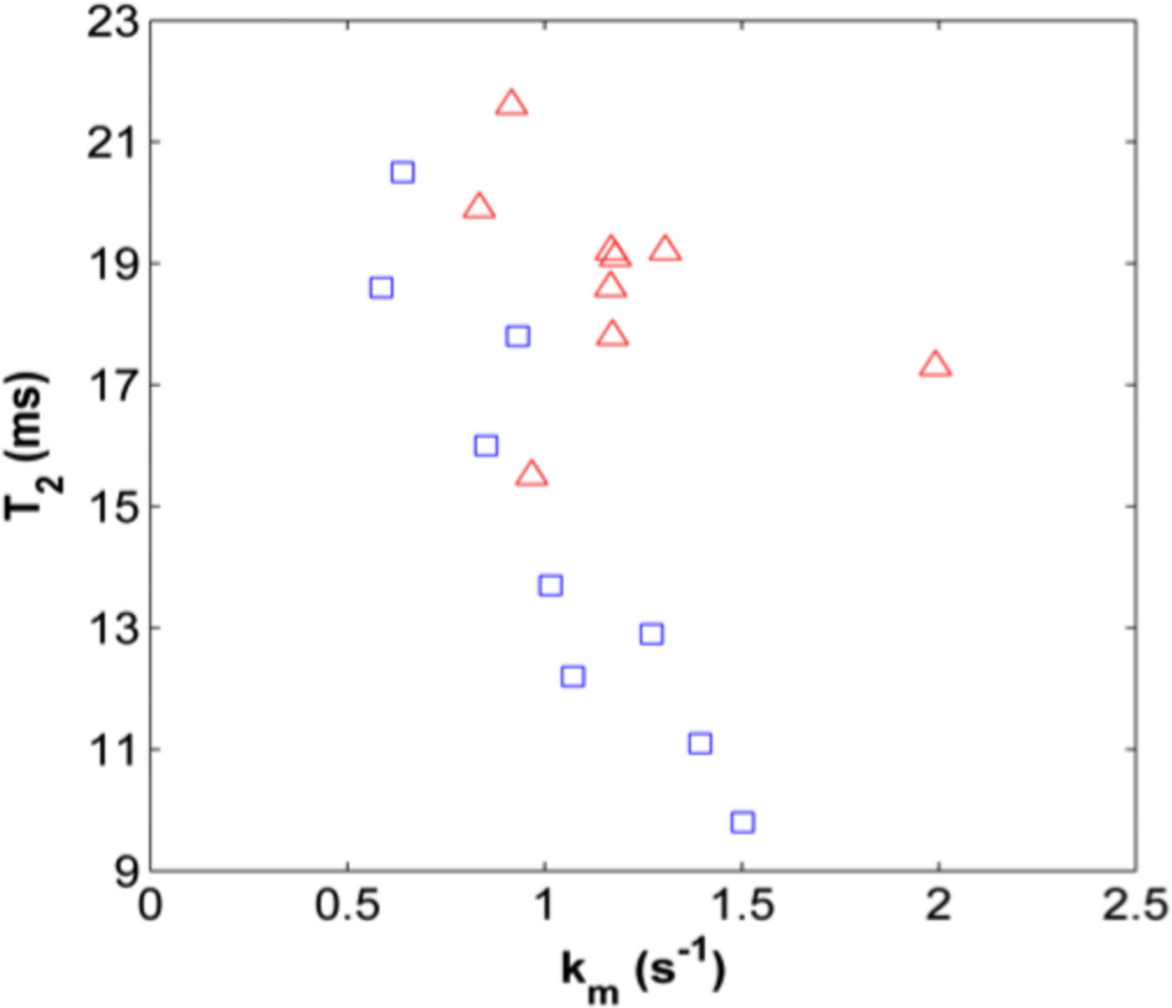
Bivariate scatter plot of T_2_ (ms) and k_m_ (s^-1^) data. Control subjects are represented as blue squares and OI patients are represented with red triangles. The projections of the data onto the respective axes recapitulate the univariate plots shown in Fig 3B and 3C. Note the decreased parameter overlap as compared to the univariate data plots.

Table 3 shows the multivariate SVM classification accuracies using combinations of the MR parameters. Six different parameter combinations exceeded the highest univariate accuracy, which was achieved using T_2_ alone. In all of these combinations, T_2_ and k_m_ were necessary parameters for optimal classification accuracy. Multiparametric analysis using the parameter set {T_2_, k_m_} resulted in 89% accuracy, the highest of any combination explored. Fig 5 is a bivariate plot of the T_2_ (ms) and k_m_ (s^-1^) values for all subjects, and shows the greatest degree of separation between the control and OI classes as compared to the respective univariate parameter plots (Fig 3). The addition of a third or fourth parameter to the {T_2_, k_m_} combination decreased the classification accuracy, with the combinations {k_m_, T_1_, T_2_} and {MTR, k_m_, T_2_, T_1_} resulting in 83% classification accuracy, and {MTR, k_m_, T_2_}, {k_m_, T_2_, ADC} and {MTR, k_m_, T_2_, ADC} resulting in a further slight decrease in accuracy to 78%.

**Table 3:** Support vector machine classification results for all MR parameter combinations.

MR Parameter combinations*		Training set			Test Set	
	Sensitivity	Specificity	Accuracy	Sensitivity	Specificity	Accuracy
MTR/k_m_	1.0	1.0	1.0	0.44	0.44	0.44
MTR/T_2_	1.0	0.90	0.95	0.67	0.56	0.61
MTR/T_1_	0.59	0.89	0.74	0.44	0.78	0.61
MTR/ADC	1.0	1.0	1.0	0.33	0.33	0.33
k_m_/T_2_*	0.89	0.99	0.94	0.89	0.89	0.89
k_m_/T_1_	0.72	0.76	0.74	0.44	0.56	0.50
k_m_/ADC	1.0	1.0	1.0	0.67	0.67	0.67
T_2_/T_1_	0.90	0.56	0.73	0.78	0.56	0.67
ADC/T_2_	1.0	1.0	1.0	0.56	0.33	0.44
T_1_/ADC	0.62	0.75	0.69	0.33	0.67	0.50
MTR/k_m_/T_2_*	0.89	0.99	0.94	0.78	0.78	0.78
MTR/k_m_/T_1_	1.0	1.0	1.0	0.22	0.0	0.11
MTR/k_m_/ADC	1.0	1.0	1.0	0.33	0.22	0.28
MTR/T_2_/T_1_	1.0	0.67	0.84	0.78	0.56	0.67
MTR/T_2_/ADC	1.0	1.0	1.0	0.67	0.44	0.56
MTR/T_1_/ADC	1.0	1.0	1.0	0.11	0.22	0.17
k_m_/T_2_/T_1_*	0.89	0.90	0.90	0.89	0.78	0.83
k_m_/T_2_/ADC*	0.88	0.95	0.92	0.78	0.78	0.78
k_m_/T_1_/ADC	0.85	0.60	0.73	0.56	0.33	0.44
T_2_/T_1_/ADC	0.99	0.58	0.78	0.78	0.56	0.67
MTR/k_m_/T_2_/T_1_*	0.89	0.89	0.89	0.89	0.78	0.83
MTR/k_m_/T_2_/ADC*	0.89	0.92	0.90	0.78	0.78	0.78
MTR/k_m_/T_1_/ADC	1.0	1.0	1.0	0.33	0.22	0.28
MTR/T_2_/T_1_/ADC	1.0	0.69	0.84	0.67	0.56	0.61
k_m_/T_2_/T_1_/ADC	0.92	0.85	0.89	0.78	0.56	0.67
MTR/k_m_/T_2_/T_1_/ADC	0.89	0.97	0.93	0.67	0.67	0.67

* Asterisk indicates multiparametric combinations that improved upon the highest univariate test accuracy

Several parameter combinations including, {MTR, k_m_}, {MTR, ADC}, {T_2_, ADC}, {MTR, k_m_, T_1_}, {MTR, k_m_, ADC}, {MTR, T_1_, ADC}, {k_m_, T1, ADC} and {MTR, k_m_, T_1_, ADC}, yielded results of limited quality with classification accuracies below 50%, that is, worse than random. Each of these multiparametric combinations included at least one parameter with poor univariate classification accuracy (Table 2).

## Discussion

MRI has been used extensively to assess the composition of collagenous tissues [17, 27–30]. Here, we demonstrate that straightforward classification techniques based on quantitative MR imaging can be utilized to discriminate skin from OI and unaffected subjects, even though samples are similar in their histological appearance. Several MR parameters, including those explored in the present study, have been shown to be sensitive to changes within the layers of the skin [30–33]. Bittoun *et al*. [17] used MRI to investigate age-related effects and found that ADC increased in the dermis of skin of older subjects, whereas differences between T_1_ and T_2_ were more variable. In a separate study, the same group found that proton density increased in the dermis of aged skin, which they attributed to a decrease in macromolecular content [31]. In general, MR skin studies have interpreted changes in magnetization transfer, T_1_ and ADC as reflective of water less tightly bound to collagen, and of variations in tissue hydration and macromolecular content [29]. Since compromised collagen quantity and integrity are characteristic of OI tissues, it is not surprising that combinations of these MR parameters, particularly those incorporating T_2_ and k_m_, successfully detected changes within skin from OI patients. The finding of significant trends of decreasing MTR, decreasing k_m_, and increasing T_2_ with increasing age in the control group is consistent with previous findings in other collagenous tissues [34–36] and indicates the expected loss of tissue organization with age. No comparable correlations were seen in the OI subjects, for which these MR parameters displayed no trends with respect to age. This indicates that the effect on MR parameters of the macromolecular manifestations of OI are much stronger than the relatively weak, though statistically significant, effects of age. However, there are limited studies quantitating these effects in skin [17, 31, 37].

To our knowledge, there are no MR studies of skin in patients with type I collagen mutations. We previously demonstrated the potential for MR to differentiate phenotypic differences in the skin of *oim* homozygous, *oim* heterozygous and control mice [16]. T_2_ and k_m_ maps were acquired and were found to distinguish various layers of the skin. Longer T_2_ values were reported in the dermis of both the homozygous and heterozygous animals. We postulated that T_2_ is lengthened in this layer because the *oim* mutation compromises the dermis, leading to greater hydration due to lower collagen content or impaired collagen fibril packing. Nevertheless, the present study expands on this by applying additional MR parameters and multivariate analysis to the quantitative analysis of human skin. Our results support the use of MR imaging to distinguish skin with and without collagen-related mutations.

Quantitative univariate MR studies of other connective tissues, such as cartilage, have shown modest success in classifying samples as healthy or degraded based on differences in individual parameters [19]. However, the substantial overlap of the individual MR parameter values observed for healthy and degraded cartilage limited the specificity of these analyses. The limited connection between statistically significant differences and discriminant properties of a measured variable has been pointed out previously[38]. Markedly improved classification accuracy was achieved through the use of multivariate analysis. Here, we report similar findings in skin; univariate classification accuracies ranged from 39–67% and were limited by overlapping mean parameter values between control and OI groups. When multivariate analysis was performed, SVM classification of OI and control samples yielded accuracies up to 89%, using as few as two MR parameters.

For all combinations explored in this study, T_2_ and k_m_ were necessary parameters for maximum accuracy in classifying skin samples. T_2_ measurements have been reported to be sensitive to free and bound water content in the skin [30], where increased water mobility, which is expected in OI tissues with compromised extracellular matrix integrity, results in longer measured T_2_ values. The magnetization transfer rate is dependent on collagen-cross linking, with increased cross-linking leading to increased values for k_m_ [34]. In addition, a decrease in k_m_ is generally associated with decreased collagen and elastin content [39, 40], also characteristic of OI mutations [41].

The multivariate quantitative MRI approach presented here may show promise for clinical application. For milder OI phenotypes that present with skeletal fractures but lack the other clinical hallmarks of OI, the use of MRI could be an ideal method for confirmation of disease. This would be especially helpful in cases of suspected non-accidental injury. While our results were not selective for specific OI types, traditional type I collagen defects ranging from mild to more severe (types I-IV OI) and type IX OI, which results from a defect in the folding and chaperoning of collagen molecules [7], were included.

In spite of these encouraging results, there were several limitations to this study. The classification models would likely be improved by including data from additional samples, which would increase the training dataset size to stabilize the models. Nevertheless, we did implement the leave-one-out analysis, a conventional approach to class analysis for small datasets [25], and our present results may motivate a larger-scale study. Even so, large sample sizes will be difficult to obtain for this rare disease. For these reasons, it is premature to draw definitive conclusions about the utility of this approach in the clinical setting. Regardless, it is notable that the presence of OI can be detected with reasonable accuracy based on noninvasive readily available MR imaging parameters.

Another limitation is that the MRI data were acquired at 9.4T under optimal experimental conditions at high resolution. While this served to establish proof of principle, further studies performed under more clinically-relevant conditions, including at a field strength of 3T, at body temperature, and without the use of biopsies, would be necessary to explore translation to the clinical setting. Our data acquisition was lengthy, ranging from 44 minutes to 5 hours to obtain high-resolution maps of all parameters. However, with relatively homogeneous parameter values having been obtained throughout the dermis, the resolution requirement may be greatly relaxed. In addition, while the optimal combination of MR parameters remains to be definitively established, classification according to only two, or at most three, parameters, may be achievable, resulting in a greatly decreased exam time. Finally, we explored multivariate analysis using the SVM; many other methods are available [26]. However, the SVM is particularly suitable for this small dataset as it does not require any assumptions about the underlying statistical structure of the data.

In summary, quantitative MRI can be used to discriminate between human OI and control skin samples using standard multivariate statistical techniques. Translation of this methodology to a clinical setting may enable the design of a rapid, non-invasive modality for diagnosis of OI as a supplement to traditional diagnostic methods, which would be especially beneficial in suspected cases of non-accidental injury.

## Supporting Information

S1 Table MRI Parameters from OI and Control Skin Biopsies. (XLSX)
